# Contribution of SERCA and IP3 sensitivity to calcium signaling in astrocytes: a computational study

**DOI:** 10.1186/1471-2202-12-S1-P201

**Published:** 2011-07-18

**Authors:** Eeva Toivari, Katri Hituri, Tiina Manninen, Tuula O Jalonen, Marja-Leena Linne

**Affiliations:** 1 Department of Signal Processing, Tampere University of Technology, P.O. Box 553, FI-33101 Tampere, Finland; 2 Department of Physiology and Neuroscience, St. George’s University, School of Medicine, Grenada, West Indies

## 

Modeling the mechanisms of astrocytic calcium signals is important, as astrocytes have an essential role in regulating the neuronal microenvironment of the central nervous system [1,2]. The results of the wet-lab and clinical studies can be complemented by mathematical models to gain better understanding of the complex molecular level interactions seen, for example, in the pathogenesis of Alzheimer’s disease (AD). In the aging brain astrocytes are known to change their phenotype [3], also their ionic equilibrium and function can be altered by the interaction of released and accumulated transmitters and peptides, such as, amyloid-β peptides [Aβ, 4]. The authors have recently shown, experimentally and computationally, that small amounts of Aβ25-35 fragment amplify the transmitter-induced calcium signals in astrocytes [5]. The reason for the amplification may be changes in calcium release from endoplasmic reticulum (ER) via, for example, changes in the function of sarco(endo)plasmic calcium adenosine 5’-triphosphatase (SERCA) pumps and/or in intracellular inositol 1,4,5-trisphosphate (IP3) sensitivity [6]. Mutations in presenilin 1 (one of the factors in familial AD involved in the accumulation of Aβ fragments in the brain) may change the activity of the SERCA, which pumps the cytosolic calcium into the ER lumen, leading eventually to higher concentration of calcium in ER [6]. Thus, the current hypothesis is that exceptional cytosolic calcium signals via ER, overfilled with calcium, may explain the calcium changes detected in the presence of Aβ.

We here study the effect of SERCA pumps and IP3 sensitivity on calcium signals in astrocytes by further exploring the existing deterministic [7] and stochastic [5] models to explain the altered calcium regulation. The models include the six major mechanisms known to be involved in calcium signaling in astrocytes; 1) calcium leak from/to extracellular matrix (ECM), 2) capacitive calcium entry from ECM, 3) calcium entry via ionotropic receptors, 4) calcium leak from intracellular stores, such as ER, 5) storage of calcium to ER via SERCA pumps, and 6) calcium release from ER mediated by IP3. In this study, we computationally explore and verify the role of SERCA pump and IP3 sensitivity -induced changes in intracellular calcium signals experimentally shown in [8] and [9]. The understanding of calcium signals in astrocytes is essential as the changes in astrocytic calcium signaling are prone to cause widespread alterations in neuronal network function and can lead to neurological disorders.

## References

[B1] Kimelberg HK Jalonen T Walz W S. Murphy Regulation of the Brain Microenvironment: Transmitters and Ions Astrocytes: Pharmacology and Function 1993 Academic Press Inc. 193 228

[B2] Seifert G Schilling K Steinhäuser C Astrocyte dysfunction in neurological disorders: a molecular perspective Nature 2006 7 194 206 10.1038/nrn1870 16495941

[B3] Simpson JE Ince PG Lace G Forster G Shaw PJ Matthews F Savva G Brayne C Wharton SB Astrocyte phenotype in relation to Alzheimer-type pathology in the ageing brain Neurobiol. Aging 2010 31 578 590 10.1016/j.neurobiolaging.2008.05.015 18586353

[B4] Casley CS Lakics V Lee H Broad LM Day TA Cluett T Smith MA O'Neill MJ Kingston AE Up-regulation of astrocyte metabotropic glutamate receptor by amyloid-β peptide Brain Res 2009 1260 65 75 10.1016/j.brainres.2008.12.082 19401173

[B5] Toivari E Manninen T Nahata AK Jalonen TO Linne ML Effects of Transmitters and Amyloid-beta Peptide on Calcium Signals in Rat Cortical Astrocytes: Fura-2AM Measurements and Stochastic Model Simulations Accepted to PLoS ONE 10.1371/journal.pone.0017914 PMC3066169 21483471

[B6] Leissring MA Akbari Y Fanger CM Cahalan MD Mattson MP LaFerla FM Capacitative calcium entry defi-cits and elevated luminal calcium content in mutant presenilin-1 knockin mice J. Cell Biol 2000 149 793 797 10811821 10.1083/jcb.149.4.793 PMC2174559

[B7] Di Garbo A Barbi M Chillemi S Alloisio S Nobile M Calcium signalling in astrocytes and modulation of neural activity BioSystems 2007 89 74 83 10.1016/j.biosystems.2006.05.013 17196325

[B8] Green KN Demuro A Akbari Y Hitt BD Smith IF Parker Ian LaFerla FM SERCA pump activity is physio-logically regulated by presenilin and regulates amyloid β production J. Cell Biol 2008 181 7 1107 1116 18591429 10.1083/jcb.200706171 PMC2442205

[B9] Morita M Kudo Y Growth Factors Upregulate Astrocyte [Ca^2+^]_i_ Oscillation by Increasing SERCA2b Expression Glia 2010 58 1988 1995 10.1002/glia.21067 20878766

